# Correction to “XUV Yield Optimization of Two‐Color High‐Order Harmonic Generation in Gases”

**DOI:** 10.1002/nap2.70146

**Published:** 2026-06-05

Raab, A.‐K., Redon, M., Roscam Abbing, S., Fang, Y., Guo, C., Smorenburg, P., Mauritsson, J., Viotti, A.‐L., L'Huillier, A. and Arnold, C.L. (2025), XUV yield optimization of two‐color high‐order harmonic generation in gases. *Nanophotonics*, 14: 3947–3953. https://doi.org/10.1515/nanoph‐2024‐0579.

In the originally published version of this manuscript, the “Research Funding” section was incomplete. The following sentence was missing:

“Y.F. is supported by the European Union's Horizon Europe research and innovation programme under the Marie Skłodowska‐Curie grant agreement No. 101153960.”

The complete “Research Funding” section should read:

“The authors acknowledge support from the Swedish Research Council (Grant Nos. 2013‐8185, 2021‐04691, 2022‐03519, and 202304603), the European Research Council (advanced grant QPAP, Grant No. 884900), the Crafoord Foundation, and the Knut and Alice Wallenberg Foundation. A.L.H. is partly supported by the Wallenberg Center for Quantum Technology (WACQT) and funded by the Knut and Alice Wallenberg Foundation. Y.F. is supported by the European Union's Horizon Europe research and innovation programme under the Marie Skłodowska‐Curie grant agreement No. 101153960.”

We apologize for this error.

